# The Gap in Medical Informatics and Continuing Education Between the United States and China: A Comparison of Conferences in 2016

**DOI:** 10.2196/jmir.8014

**Published:** 2017-06-21

**Authors:** Jun Liang, Kunyan Wei, Qun Meng, Zhenying Chen, Jiajie Zhang, Jianbo Lei

**Affiliations:** ^1^ Second Affiliated Hospital School of Medicine,Zhejiang University Hangzhou China; ^2^ Department of Gastroenterology Affiliated Hospital of Southwest Medical University Luzhou China; ^3^ Center for Statistics and Information National Health and Family Planning Commission of China Beijing China; ^4^ Library of Zhejiang University Hangzhou China; ^5^ School of Biomedical Informatics University of Texas Health Sciences Center Houston, TX United States; ^6^ Center for Medical Informatics Peking University Beijing China; ^7^ School of Medical Informatics and Engineering Southwest Medical University Luzhou China

**Keywords:** medical informatics, conferences, continuing education, Sino-American comparison

## Abstract

**Background:**

China launched its second health reform in 2010 with considerable investments in medical informatics (MI). However, to the best of our knowledge, research on the outcomes of this ambitious undertaking has been limited.

**Objective:**

Our aim was to understand the development of MI and the state of continuing education in China and the United States from the perspective of conferences.

**Methods:**

We conducted a quantitative and qualitative analysis of four MI conferences in China and two in the United States: China Medical Information Association Annual Symposium (CMIAAS), China Hospital Information Network Annual Conference (CHINC), China Health Information Technology Exchange Annual Conference (CHITEC), China Annual Proceeding of Medical Informatics (CPMI) versus the American Medical Informatics Association (AMIA) and Healthcare Information and Management Systems Society (HIMSS). The scale, composition, and regional distribution of attendees, topics, and research fields for each conference were summarized and compared.

**Results:**

CMIAAS and CPMI are mainstream academic conferences, while CHINC and CHITEC are industry conferences in China. Compared to HIMSS 2016, the meeting duration of CHITEC was 3 versus 5 days, the number of conference sessions was 132 versus 950+, the number of attendees was 5000 versus 40,000+, the number of vendors was 152 versus 1400+, the number of subforums was 12 versus 230, the number of preconference education symposiums and workshops was 0 versus 12, and the duration of preconference educational symposiums and workshops was 0 versus 1 day. Compared to AMIA, the meeting duration of Chinese CMIAAS was 2 versus 5 days, the number of conference sessions was 42 versus 110, the number of attendees was 200 versus 2500+, the number of vendors was 5 versus 75+, and the number of subforums was 4 versus 10. The number of preconference tutorials and working groups was 0 versus 29, and the duration of tutorials and working group was 0 versus 1.5 days.

**Conclusions:**

Given the size of the Chinese economy and the substantial investment in MI, the output in terms of conferences remains low. The impact of conferences on continuing education to professionals is not significant. Chinese researchers and professionals should approach MI with greater rigor, including validated research methods, formal training, and effective continuing education, in order to utilize knowledge gained by other countries and to expand collaboration.

## Introduction

### Background

China, the world’s second largest economy, launched its second health reform in 2010 with considerable investments in medical informatics (MI) [1]. An important contribution of this reform is its definition of MI as one of the “four constructs and eight pillars,” or foundations of health care reform [2]. Under the guidance and incentives provided in a series of policies, the Chinese government and health care informatics industry have invested a huge amount of money in hospital informatics and population health informatics. However, current MI in China can be characterized as “hot in industry application, and cold in academic research” [3]. To the best of our knowledge, research on the outcomes of this ambitious health reform has been limited.

Conferences including tutorial sessions, talks, product exhibitions, and conference proceedings are potentially important channels of continuing education, allowing professionals to update their knowledge and learn about industry developments. Therefore, this study focused on the characteristics of mainstream MI professional conferences in China and compared them to those in the United States, in order to promote the international exchange of knowledge to improve MI in China and potentially other nations, where progress in the discipline is perceived to be lagging.

### Objectives

We focused on four national mainstream medical informatics conferences in China—the China Medical Information Association Annual Symposium (CMIAAS 2015) [4], the China Hospital Information Network Annual Conference (CHINC 2016) [5], the China Health Information Technology Exchange Annual Conference (CHITEC 2016) [6], and the China Annual Proceeding of Medical Informatics (CPMI 2016) [7]). These conferences are also the only four academic and industry conferences that are certified to offer continuing education credits by the National Commission of Health and Family Planning. We then compared the characteristics of those four conferences with two international MI conferences based in the United States—the American Medical Informatics Association (AMIA) and the Healthcare Information and Management Systems Society (HIMSS) 2016 conferences—with the aim of identifying gaps and summarizing lessons learned to encourage greater international cooperation.

## Methods

### Selection of Mainstream Medical Informatics Conferences in China

Following the eHealth Preferred Reporting Items for Systematic Reviews and Meta-Analysis (PRISMA) methodology [8], we chose four MI conferences as the mainstream conferences in mainland China: (1) CMIAAS hosted by the China Medical Informatics Association (the only country representative of International Medical Informatics Association); (2) CPMI hosted by the Medical Informatics Branch, Chinese Medical Association, which is the most authoritative organization for Chinese medicine; (3) CHINC hosted by the Committee on Information Management, Chinese Hospital Association; and (4) CHITEC hosted by the China Institutes of Health Information (the largest two industry associations in MI).

### Data Collection

The three largest Chinese literature databases were searched: the VIP database of Chinese scientific and technical journals [9], the Chinese National Knowledge Infrastructure [10], and the Wanfang data retrieval platform [11]. The databases were searched for “conference proceedings”. Keywords included the Chinese name of the conferences; the Chinese organizer of the conferences (China Medical Informatics Association, the Committee on Information Management, Chinese Hospital Association, China Institutes of Health Information, and the Medical Informatics Branch, Chinese Medical Association); and the English acronym of each conference (CMIAAS, CHINC, CHITEC, and CPMI). Conference proceedings between 1985 and 2015 were retrieved. In addition, we searched for relevant supporting information and biographical references, including the purpose, scope of business, and “call for papers” related to each conference.

Data for AMIA and HIMSS—the two American MI conferences—were extracted from literature reviews [12,13], along with the “letter of welcome” for AMIA and HIMSS and website information regarding conference organizers [14,15].

### Data Extraction

We exported information on target conferences from the literature and databases, including the following:

The scale of the conference, including the name of the conference, the organizer, its year of inception, its schedule, the duration of the conference, and the number of attendees.

General information regarding the proceedings, including title, publication year, author affiliation, the name of the conference, and the author’s geographic area (extracted using a self-developed tool).

Topics of conference proceedings, which we collected and analyzed, as well as the “call for papers” of the CMIAAS, CHINC, CPMI, and CHITEC (2000-2016) and the organizer’s scope of business.

We used EndNote X2, MS Excel 2011, and Python 2.7 for data processing and analysis.

## Results

To further investigate the current level of MI academic research and health information technology (HIT) applications in China, we compared the number of conference sessions, number of attendees, topics presented in conference proceedings, academic research, real-world applications, presentations of academic achievement, number of participating companies, and review criteria among four Chinese mainstream medical informatics conferences, as well as two US conferences. The results are shown in Tables 1 and 2, and specific details are presented in Table 3. The topics of the conference proceedings indicated that these conferences focused on highly similar topics: among them, 10 research fields, including but not limited to, hospital informatics; regional, public, and grassroots health informatics; and telemedicine.

**Table 1:** Comparison of major characteristics of MI conferences (academic) in United States and China, 2016.

Characteristics	CMIAAS 2015	CPMI 2016	AMIA 2016
Days of meeting, n	2	2	5
Conference sessions, n	42	83	110
Attendees, n	200	300	2500
Participating companies, n	5	17	75
Overall volume of academic achievements^a^	71	248	1349
Presentations of academic achievements, n	1	2	6
Subforums, n	4	4	10
Topics in conference proceedings, n	6	5	20
Pre-symposium tutorials & work group sessions, n	0	7	29
Days of pre-symposium tutorials & working group, n	0	0.5	1.5

^a^Since CPMI 2016 conference papers were still not available from the three major Chinese literature databases (the VIP database of Chinese scientific and technical journals, the Chinese National Knowledge Infrastructure, and the Wanfang database) at the time of writing, CPMI 2015 conference papers were cited here instead.

**Table 2:** Comparison of major characteristics of MI conferences (industry) in United States and China, 2016.

Characteristics	CHINC 16	CHITEC 16	HIMSS 16
Days of meeting, n	3	3	5
Sessions, n	178	132	950
Attendees, n	3500	5000	40,000
Participating companies, n	138	152	1400
Overall volume of conference proceedings^a^	853	667	0
Concurrent education sessions, n	64	40	300
Subforums, n	23	12	230
Topics in conference proceedings, n	6	10	20
Pre-symposium tutorials & workshops, n	4	0	12
Days of pre-symposium tutorials & working groups, n	1	0	1

^a^Since the conference papers of CHINC 2016 and CHITEC 2016 were still not available from the three major Chinese literature databases (the VIP database of Chinese scientific and technical journals, the Chinese National Knowledge Infrastructure, and the Wanfang database) at the time of writing, the conference papers of CHINC 2015 and CHITEC 2015 were cited here instead.

**Table 3:** Summary of CMIAAS 2015, CHINC 2016, CHITEC 2016, CPMI 2016, AMIA 2016, and HIMSS 2016.

Characteristics	CMIAAS 2015	CPMI 2016	AMIA 2016	CHITEC 2016	CHINC 2016	HIMSS 2016
Organizer	China Medical Informatics Association	Medical Informatics Branch, Chinese Medical Association	American Medical Informatics Association	China Institutes of Health Information	Committee on Information Management, Chinese Hospital Association	Healthcare Information and Management Systems Society of the US
Inception	1981	1993	1977	2004	1997	1962
Schedule	Every 3 years	Annual	Annual	Annual	Annual	Annual
Meeting duration	1 day	2 day	5 days	3 days	3 days	5 days
Attendees, n	200+	300+	2500+	5000	3500+	40,000+
Composition of attendees	Medical institutions, universities, research institutes	Medical institutions, research institutes, universities	Medical institutions, universities, research institutions, companies	Medical institutions, research institutes, government authorities, enterprises, some universities	Medical institutions, enterprises, some universities and research institutes	Companies, medical institutions, research institutions
Academic achievement	4 forums, 71 conference papers	4 forums, half-day preconference seminar, 248 conference papers, including 13 papers presented at general conference and 48 papers at forums^a^	10 forums, one and half-day preconference seminar, 29 continuing education sessions, and 110 lectures; 151 full-text papers, 50 student articles, 1050 posters, 11 system presentations, 80 abstracts, 7 contests of student-led project design. Rigorous peer review mechanism.	12 forums, 40 continuing education lectures; 667 conference papers, of which 37 were nominated as “outstanding papers”^a^	23 forums, 64 continuing education lectures, 1-day preconference seminar; 853 conference papers, of which 71 were nominated as “outstanding papers”^a^	˃230 forums and presentations, ˃300 concurrent education sessions, 1-day preconference seminar
Paper review mechanism	Format review only	Format review only	Rigorous peer review mechanism	Format review only	Format review only Presenters required to submit abstracts and PPT for peer review	
Participating companies, n	5	17	75	152	138	1400+, with review performed between 12 regions
Role of participants	Institution IT staff (48%), university research staff (33%), MI specialists (7%)	Institution IT staff (34%), university research staff (27%), MI specialist (24%)	MDs (27%), research staff (19%), institution IT and administrative staff (17%)	Institution IT staff (57%), government functional personnel, students (29%), MI specialists (6%)	Institution IT staff (78%), software providers, R&D staff (14%), university research staff (5%)	Medical institution leaders (25%), software providers, R&D staff (23%), government functional personnel, students (13%)
Fields covered^b^	B, D, I, F, P, T	L, D, B, F, P	A, B, C, D, E, F, G, H, I, J, K, L, M, N, O, P, Q, R, S, T	M, N, K, C, D, I, F, H, Q, T	B, D, I, F, P, T	A, B, C, D, E, F, G, H, I, J, K, L, M, N, O, P, Q, R, S, T

^a^Since the conference papers of CHINC 2016, CHITEC 2016 and CPMI 2016 were still not available from the three major Chinese literature databases (the VIP database of Chinese scientific and technical journals, the Chinese National Knowledge Infrastructure, and the Wanfang database) at the time of writing, the conference papers of CHINC 2015, CHITEC 2015, and CPMI 2015 were cited here instead.

^b^A: consumer health informatics; B: clinical information management; C: decision support system; D: electronic medical records; E: medical language processing; F: nursing informatics; G: achievement evaluation; H: public health informatics: I: information retrieval; J: medical cognitive science; K: clinical project management; L: computer-based training; M: coding, classification, and terminology; N: clinical guidelines for computerization; O: image, robotics, virtual medical treatment; P: signal processing; Q: standards, social, and legal issues; R: dental informatics; S: artificial intelligence; T: telemedicine.

We briefly analyzed the data shown in Table 3 and reached the following primary conclusions regarding the current status of MI development in China and the United States.

First, for academic exchange conferences, we compared the relatively large CPMI conference with AMIA (the annual symposium 2016) and observed that the number of attendees and number of conference papers of the CPMI were approximately only 8% of the numbers of AMIA conference papers. For presentation formats of academic achievements, at AMIA 2016, academic achievements were presented through various means—including full-text papers, posters, student papers, abstracts, design contests, system demonstrations, and descriptions—whereas, at CPMI 2015, academic achievements were presented only as full-text and exchange papers. Of the topics, only 7 fields were covered at CPMI 2015, of which 70.6% of papers were related to information retrieval and hospital informatics. Conversely, all 20 subfields related to MI were covered at AMIA 2016. At the same time, there was remarkable gap between the two conferences in terms of number, size, and degree of attention of the special pre-symposium continuing education sessions. AMIA 2016 scheduled 29 high-level tutorials and working groups in the day and a half before the official symposium, and the average time of each session lasted about 53 minutes. The CPMI 2016 scheduled only 7 tutorials in the afternoon 1 day before the official symposium (ie, less than a quarter of AMIA), and the average time of each session lasted only 25 minutes (ie, only half of AMIA). Therefore, Chinese professionals had fewer continuing education opportunities to attend tutorials offered by conferences. In terms of topics included in the academic conference, AMIA 2016 contained 20 themes, while CMIAAS 2015 and CPMI 2016 contained only 6 and 5, respectively. In terms of participant distribution, AMIA 2016 was mainly composed of medical doctors, researchers, and medical institutions information technology and administrative personnel, while CMIAAS 2015 and CPMI 2016 comprised mainly information technology management of medical institutions, university researchers, and professional MI researchers. Medical doctors were seldom involved.

We then examined the data for HIT application conferences where the gap was also significant. Our comparison of the relatively large CHITEC 2016 and HIMSS 2016 revealed that the number of attendees at CHITEC 2016 was only 12.5% of the number at HIMSS 2016 (5000+ compared to 40,000+), and the number of participating companies at CHITEC 2016 was only 10.9% of the number at HIMSS 2016 (152 compared to 1400+). For academic value-related measures, CHITEC 2016 included only 12 subforums and 40 continuing education lectures, which were, respectively, 5% and 13% of the forums and lectures at HIMSS 2016 (230+ forums and 300+ concurrent education sessions over 5 days). For topics, 10 fields were covered at CHITEC 2015; however, 73.8% of the conference papers were related to hospital informatics or population health informatics, whereas all 20 subfields related to MI were covered at HIMSS 2016. In addition, there was a similar gap between the two conferences in terms of number, size, and degree of attention of pre-symposium continuing learning opportunities. HIMSS 2016 scheduled 12 high-quality preconference education symposiums and workshops, while CHINC 2016 scheduled only 4 (ie, less than half of HIMSS) for 1 day before the official meeting. In terms of subjects, industry conference HIMSS 2016 contained 20 themes, while CHITEC 2016 and CHINC 2016 contained only 10 and 6, focusing mainly on hospital and clinical informatics. In terms of participant distribution, HIMSS 2016 had a similar composition of participants to that of CHITEC 2016 and CHINC 2016, but with more uniform and reasonable distribution.

Third, we noted a significant gap between the academic and continuing education value of Chinese and US MI conference proceedings papers. Specifically, at Chinese HIT conferences, only format review was conducted, due to the lack of well-rounded peer reviewers who had professional training in MI courses. As a consequence, the academic value of the Chinese conference papers was fairly low. In contrast, rigorous peer review was implemented for AMIA 2016. According to data published on its website, the acceptance rate for AMIA’s proceedings papers is less than 30%. Thus, AMIA has been recognized by the China Computer Federation Academic Committee as “an important internationally recognized conference” [16].

Overall, we believe that the huge gap between the US and Chinese MI conferences is closely related to their economic strength, population, and health care system. According to 2015 statistics, the US gross domestic product is 1.78 times the Chinese (US $18 trillion and 40 billion [17] vs US $10 trillion and 140 billion [18]). The US population is only 23% of China (314 million [19] vs 1 billion 370 million [20]), but US spending on health care accounted for 16.9% of its gross domestic product [21], which is only 5.95% for China [22]. That is to say, in the same year the health expense per capita was $9451 in the United States [21] and was only $438 in China [22]. The former is 21.6 times the latter. In addition, a huge difference between the Chinese and US health systems is that in the United States, new inventions will soon be put into use [23]. MI is a new interdisciplinary field that has a wide market prospect and naturally has been attracting a lot of investment funds support. Investors want to reduce medical cost through the use of new technologies, forcing medical institutions’ cost control under the premise of quality assurance. Therefore, it is not surprising that the number of attendees of HIMSS 2016 is 8 times that of CHITEC 2016, and AMIA 2016’s academic achievements are 8.3 times those of CPMI 2016.

In addition, this phenomenon of focusing more on application in HIT and less on research on MI has led to repeated HIT construction in China and a huge resource waste. Contrasted to the United States, there exists a difficulty in applying theoretical research to real-world problems in China, the foundation of theoretical research in medical informatics is weak, and few such studies have been published in China. At present, only a few Chinese teaching institutions have graduate courses or research institutes focused on MI. Moreover, very little investment from the Chinese government has been devoted to basic MI research compared with the huge investments into HIT application, especially MI. We searched and compared the medical information projects funded by Natural Science Foundation of China (NSFC) [24]. As the main channel from the Chinese government to support basic and applied basic research, NSFC is targeted at the whole country for researchers from universities, colleges, research institutions, and enterprises. NSFC is more or less similar to the US National Institutes of Health (NIH). The funded area directly related to MI in NSFC was very limited, with only one domain code set up for independent medical information from 2005, which is code H1814 (medical information system and telemedicine). The number of MI projects funded by NSFC was extraordinarily limited compared to NIH. In 2015, only 3 MI projects (under code H1814) over 3 institutions were funded, with a total amount of USD $284,000; the amount per project was USD $4700. This was in sharp contrast to NIH where 122 MI projects over 82 institutions were funded, with a total of USD $6,514,279. The amount per project was USD $545,199, which were 40.7 times, 27.3 times, 234.2 times, and 5.76 times the Chinese counterparts respectively in 2015. In summary, compared with their American counterparts, funding from government in terms of quantity and amount in basic MI research was very poor in China. The results are shown in Table 4.

**Table 4:** Comparison of funding support for the academic areas of MI by major funders: NSFC (China) and NIH (US), 2015.

Organization	Funded projects, n	Funded institutions, n	Average amount (USD)	Total amount of funded projects (USD)
NSFC	3	3	94,700	284,000
NIH	122	82	545,199	66,514,279

## Discussion

### Principal Findings

This cross-sectional study of the quantitative measures of Chinese and US academic and industry exchange conferences showed a large gap between China and the United States in both MI academic research and HIT applications. In addition, there exists a gap in the effect of conferences themselves and proceedings on continuing education to MI researchers and professionals.

Our research showed that MI conferences in China exhibit a significant gap compared to their US counterparts in terms of scale, quality, timeliness, breadth, and depth, from the perspective of discipline development and continuing education. The value of conferences as a continuing education channel to MI professionals in China is also far less than their US counterparts. The training tutorials, lectures, forums, and proceedings of MI conferences in China are more focused on HIT applications and less on the level of academic research.

### Strengths and Limitations

This study compared the development of Sino American medical informatics from the perspective of relevant conferences in the two countries. However, the measures to evaluate an overall discipline or industry development are complex. Conferences and meetings are only one part of the field. Furthermore, some information was publically unavailable, such as the demographic information of actual attendees at the Chinese MI conferences or the Chinese participants in the US MI conferences. In addition to conferences, future research should examine capital investment, published papers, patent applications, safety improvement, patient satisfaction, etc, to evaluate the outcomes of MI development in China.

### Conclusion

This study demonstrated a significant gap in MI development and continuing education between China and the United States from the perspective of conferences and analyzed the underlying reasons for this gap. There is an urgent need to elevate MI in China in the area of academic discipline and research. Relevant MI professionals need to strengthen mutual understanding and develop pertinent and effective cooperation between China and the United States as well as other global colleagues.

## Abbreviations

AMIA: American Medical Informatics Association
CHINC: China Hospital Information Network Annual Conference
CHITEC: China Health Information Technology Exchange Annual Conference
CMIAAS: China Medicine Information Association Annual Symposium
CPMI: China Annual Proceeding of Medical Informatics
HIMSS: Healthcare Information and Management Systems Society
HIT: health information technology
MI: medical informatics
NIH: National Institutes of Health
NSFC: Natural Science Foundation of China
PRISMA: Preferred Reporting Items for Systematic Reviews and Meta-Analysis
